# DNA Vaccines: Recent Developments and the Future

**DOI:** 10.3390/vaccines2040785

**Published:** 2014-10-27

**Authors:** Britta Wahren, Margaret A. Liu

**Affiliations:** 1 Department of Microbiology, Tumor and Cell Biology, Karolinska Institutet, Nobel’s Rd 16, 171 77 Stockholm, Sweden; E-Mail: Britta.Wahren@ki.se; 2 ProTherImmune, Lafayette, CA 94549, USA

**Keywords:** DNA vaccines, RNA, immune responses, clinical trials, veterinary applications, infectious diseases, cancer, immunotherapy

## Abstract

This special issue is focused on DNA vaccines, marking the two decades since the first demonstration of pre-clinical protection was published in Science (Ulmer *et al*.; Heterologous protection against influenza by injection of DNA encoding a viral protein. 1993). This introductory article provides an overview of the field and highlights the observations of the articles in this special issue while placing them in the context of other recent publications.

## 1. Introduction 

In the years following the first publication [1] of pre-clinical protection by a DNA vaccine against challenge by an infectious agent, the efficacy and immunologic mechanisms were demonstrated in a number of pre-clinical models for a variety of types of diseases, including infectious diseases, cancer, autoimmunity and allergies [2]. The appeal of the technology was because of many of its characteristics: the ability to induce both cellular and humoral immunity, the lack of the risk or of potential risk associated with certain attenuated pathogens (e.g., oral polio, or an inactivated HIV vaccine where incomplete inactivation during manufacture could be a concern), the ability to have a generic manufacturing process, and the potential for usage in low-resource settings due to all of the above. In addition, DNA vaccines provided a useful laboratory tool, such as for making both polyclonal and monoclonal antibodies, based on the ease of making plasmids encoding even transmembrane proteins (*vs.* the former need to purify the protein antigen, or make it recombinantly, then purify it, with the challenges of ensuring correct mammalian post-translational modifications, and the inability to produce soluble transmembrane proteins).

Several DNA vaccines have been licensed for veterinary applications, including an equine vaccine for West Nile Virus in 2005, a fish (salmon) vaccine against infectious hematopoietic necrosis virus in 2005, and a therapeutic canine vaccine for melanoma conditionally approved in 2007, with full licensure in 2010. Plasmid DNA encoding Growth Hormone Releasing Hormone delivered with electroporation was licensed in 2007 for pigs as a gene delivery application.

However, the human clinical potency of DNA vaccines has been overall disappointing, with no human vaccines licensed to date. Certain vaccines have resulted in strong human immune responses in early phase clinical trials. Examples include: a West Nile Virus vaccine [3] and Cytomegalovirus. For the latter, both humoral and cellular responses were seen in significant proportions of both CMV positive and negative patients in a phase II clinical trial of the CMV DNA vaccine [4] now in a Phase III efficacy trial. A regimen of DNA priming with subsequent heterologous boost with various viral vectors: Adenovirus 5, Modified Vaccinia Ankara (MVA) or Cytomegalovirus [5,6,7] resulted in strong immune responses against encoded HIV antigens. In a twist of the usual concept of induction of adaptive immunity, a human Phase II clinical trial of a DNA vaccine encoding proinsulin, when given to patients with Type 1 Diabetes, gave early indications of efficacy with increased insulin levels (as measured by the C-peptide) via a mechanism of reducing the CD8^+^ T cells that attack the insulin-producing beta cells of the pancreas [8]. To accomplish this, the DNA vaccine was constructed to minimize the CpG-mediated activation of the innate immune system (see below) by modifying CpGs to GpGs. Clinical trials of DNA vaccines are in progress for many diseases including HIV, hepatitis C, malaria, influenza, tuberculosis, diabetes, and cancer (colo-rectal, prostate, melanoma); see Table 1, and *clinicaltrials.gov* [9], which lists close to 900 DNA vaccine clinical trials. These include plasmid constructs used as a prime for boosting with other types of vaccines.

The many different and incremental improvements for DNA vaccines are well-summarized and beautifully diagrammed in the article in this special issue by Felber and colleagues [10]. The varied approaches ranging from changes in the plasmid construct, inclusion of molecular adjuvants, and different delivery technologies and sites are addressed in the article and will be discussed below. Other articles in this issue discuss specific applications and pre-clinical and clinical results which will be presented along with other recent examples from the literature.

## 2. Progress in Gene Vaccination

### 2.1. DNA and RNA Modifications

Constructions of DNA plasmids that increase expression of the inserted gene(s), and permit better intracellular transcription, translation, and secretion/expression, and non-antibiotic selection systems which unexpectedly increased expression were updated in the article in this issue by Williams [11]. Examples of DNA vaccines containing single genes encoding glycoprotein or glycoprotein precursor genes protected animals in models of rabies [12] and Lassa fever [13]. Vaccination against the Porcine Respiratory and Reproductive Syndrome (PRRS) [14] proved to be difficult, despite several construction attempts. Self-replicating mRNA was presented as an interesting modality [15] that takes immunization one step further from delivery of DNA, by eliminating the need to deliver the DNA into the nucleus for transcription. Minimal backbone vectors, so called micro-minicircles, are more stable to shear forces and prolong gene expression in mice [16].

### 2.2. Adjuvants, Carriers and Delivery Systems

Cytokines, chemokines, or co-stimulatory molecules can be co-delivered as genes along with the antigen gene either on the same or in a different plasmid. It has not yet been fully explored whether DNA plasmids can be concomitantly used to deliver adjuvants or cytokines with protein vaccines [17]. Activation of innate immunity can also contribute different features for cellular and humoral adaptive immune responses. Plasmid DNA, because of its bacterial origin, has a number of CpG motifs that are capable of stimulating innate immune responses via TLR9. A combination of TLR 7/8 and TLR 9 agonists (CpG DNA that does not encode antigens) eliminated tumors in mice and resulted in long-term protection by increasing the activity of CTL and NK cells [18]. The STING-TBK-1 signaling cascade has also been implicated [19]. Cellular transcription factors such as T-bed (a T box transcription factor) or Re1A (part of the NF-kappa B transcription complex), have also been effective as molecular adjuvants when co-administered with a DNA vaccine encoding HIV proteins [20]. A wide range of genes producing endogenous immunostimulatory proteins has been used to enhance the immunogenicity of DNA-encoded antigens, including GM-CSF, IL-2, IL-12, IL-15, and CD40L [21], and flagellin [22]. Local cytokine production at the site of plasmid administration and the type of cytokine adjuvant can influence the quality of innate and CD4^+^ and CD8^+^ responses. GM-CSF (derived from the relevant animal species) is effective in animal models. When used for immune activation in humans, the effect was not striking, despite clear evidence of biological activity [7]. The same has been the experience with some of the interleukin genes [23].

Delivery devices and carriers facilitate the eventual localization of DNA or RNA intact into the cellular nucleus, compartments or cytoplasm. Recent types of delivery systems include electroporation, needle free jet delivery and lipid-based carriers [24]. Devices that target intradermally have been shown to be more tolerable in humans and also more effective for the same dose of DNA than intramuscular injection [25]. An example of intradermal electroporation is shown by Mendoza *et al.* who demonstrated in guinea pigs that a plasmid encoding green fluorescent protein (GFP) expressed its protein as early as one hour after immunization, with a peak of expression at 24 h, and a duration of around 7 days [26]. During the experiment, keratinocytes had the highest expression when moving towards the skin surface, thus confirming that the plasmids move with the maturing keratinocyte layer. Finally most of the expressing cells are shed, which may be desirable for vaccine purposes. Improved immunogenicity by electroporation is shown in several species, such as in guinea pigs, mice and rabbits [13,27]. A combination of needle-free jet injection and electroporation showed the best induction of cellular and humoral immunities in mice [28] while efficacy of electroporation delivery was not significantly elevated in humans compared to needle-free jet delivery [29].

### 2.3. Adaptive Immunity

DNA vaccines were initially developed as a means to generate CTL responses without the use of viral vectors (or live viruses). DNA alone (or in prime-boost combinations), has been effective in vaccine clinical trials for various diseases for generating both CD4^+^ and CD8^+^ T cell responses. However, the HIV STEP trial (utilizing an Adenovirus vector) and the HVTN 505 trial (utilizing a DNA prime/Adenovirus vector boost) failed to demonstrate protection, despite generating cellular responses. In contrast, the RV144 Thai trial provided modest protection, but with an antibody correlate of protection. Various DNA prime-viral vector boost HIV vaccine trials have progressed to Phase II trials, with the generation of both broad and high cellular and antibody responses [7], but the clinical significance is unknown.

Concern has existed that DNA vaccines have been less effective for producing protective B cell antibody responses. However, even the first demonstrations of the protective efficacy of DNA vaccines [1,21], although focused on showing that DNA vaccines could produce cross-strain protective cellular responses, also showed the ability of DNA encoding influenza hemagglutinin to generate antibodies that mediated homologous protection against viral challenge. And as previously observed, the licensed DNA equine encephalitis vaccine mediates protection via antibodies [30], and was also found to induce protective antibodies in birds [31] and mice [30]. In a human phase I clinical trial, immunization with DNA encoding the West Nile Virus pre-membrane and envelope glycoproteins resulted in the generation of neutralizing antibodies as well as cellular responses [32].

Li *et al*. had previously demonstrated that DNA encoding LcrV could protect mice against lethal mucosal challenge with *Y. pestis* (plague), a disease in which antibody plays an important protective role. In this issue, they advanced the work by demonstrating that DNA encoding LcrV alone or followed by a protein boost, induces high-titers of antibodies and significant B cell development [33]. A difficulty with certain pathogens, such as HIV, has been the induction of neutralizing antibodies. When large amounts of HIV Env plasmids were given by electroporation in an aggressive immunization schedule intradermally, this resulted in high HIV-neutralizing titers in guinea pigs and rabbits but not in macaques [27].

Chinkungunya virus is a vector-borne alpha virus, which has been spreading from Africa to additional continents, notably the Americas and Asia. Various vaccines are under development including an MVA vector expressing structural genes, where one dose could protect mice against challenge [34]; an earlier study with a DNA vaccine was able to protect both mice and non-human primates [35].

Lassa virus is an arenavirus, and together with Ebola and Kongo-Krim, are hemorrhagic fever diseases with a high mortality rate for humans. Since live or live attenuated vaccines might be a risky vaccine modality of vaccine development, a DNA-based vaccine appears highly desirable [13]. In this first report of protection against Lassa fever using a plasmid DNA expressing a glycoprotein precursor gene, over 90% of guinea pigs survived a high-dose challenge. Intradermal delivery resulted in both higher neutralizing antibodies and fewer fever bouts.

### 2.4. Prime and Boost

The DNA prime/vector boost concept, first demonstrated by Hill and colleagues [36] has been expanded to include prime/boosts with different viral vectors (for example the recently published Phase I trial of a chimpanzee Adenovirus vector/MVA vaccine for malaria [37]). The important prime/boost concept is exemplified by Iyer *et al*. with SHIV-DNA followed by SHIV-MVA [21]. DNA priming appears to improve the outcome of boosting with either a more classical entity, such as proteins, or with vector-based vaccines. 

Presently, priming with DNA plasmids as the first component for gene-based vaccines for the prophylaxis for other infectious diseases such as hepatitis C, malaria and influenza are under evaluation. The potency is dependent upon DNA being the prime rather than the boost. While the mechanism is still not certain, it is possible that focusing the immune response on the one or few antigens generated by the plasmid gene(s), may result in potent boosting when larger amounts of proteins are produced by the viral vector in the context of the innate/inflammatory responses generated by the viral vector.

A unique approach combines delivery of live *Mycobacterium bovis* BCG together with plasmid DNA encoding a selected TB prototype antigen [38]. This combination induced stronger antibody and CD4^+^ and CD8^+^ T-cells to the plasmid-encoded antigen than did BCG alone, in mice. Interestingly, it also induced bystander effects with better responses to other BCG components; the effect was ascribed to additional cytokine activation by the plasmid DNA. 

### 2.5. Clinical Immunotherapy and Prophylaxis

#### 2.5.1. Veterinary Applications

A successful species for DNA immunization has been the fish. DNA vaccination against infectious hematopoietic necrosis virus now takes place at commercial level; the DNA vaccine was licensed in 2005 as noted above. Additionally, immunization against a salmon viral hemorrhagic septicemia virus has been demonstrated experimentally. Means to improve uptake and persistence of DNA vaccines in fish by molecular adjuvants or carriers are ongoing [39]. As described earlier, a DNA vaccine against West Nile Virus was licensed for horses in 2005. And an influenza DNA vaccine encoding hemagglutinin A generated immune responses (both homologous and heterologous) and protected against viral replication and clinical disease in ponies following homologous (H3N8) challenge [40].

#### 2.5.2. Human Studies

As early as 1998, the first published clinical study on HIV immune responses showed induction of new immune responses against early proteins Nef, Tat and Rev in HIV-infected individuals [41]. A plasmid DNA vaccine against cytomegalovirus (CMV) is in a phase III trial [42] for hematopoietic cell transplant recipients. The vaccine consists of two plasmids expressing CMV antigens glycoprotein B and phosphoprotein 65, both antigens that usually give rise to strong antibody responses in the infected individual. This CMV DNA vaccine was shown to reduce viremic episodes, to decrease the occurrence of detectable viremia, and to lengthen the period before the onset of viremia, compared to placebo in the Phase II study mentioned in the introduction [4].

An innovative trial of therapeutic immunization for HIV positive children and adults was reported [43,44]. Following HIV-DNA administration, children appeared to develop more of a CD4^+^ T-cell response while adults had a CD8^+^ related response. While small, these studies serve to demonstrate safety and moderate immunogenicity in these two groups of infected individuals. The same or a similar HIV-DNA vaccine followed by a vaccinia vector boost has permitted a 3-year long establishment of anamnestic memory in healthy humans, demonstrating the ability of this DNA prime-MVA boost regimen to produce memory responses [7,21].

Various DNA, adeno- and VSV-based Ebola vaccines encoding various genes have induced protective immunity in non-human primates [45]. The National Institutes of Health, US, following the demonstration of 10-months of protection of non-human primates by an Ebola vaccine utilizing a chimpanzee adenovirus vector with a boost has initiated Phase I safety testing of the vaccine in humans in September 2014, in the face of the enlarging uncontrollable Ebola epidemic in West Africa [46].

### 2.6. Cancer

When cancers result from infection with viruses, such as human papilloma viruses (HPV) or hepatitis B virus (HBV), it is possible to target viral proteins and by preventing infection, to decrease the incidence of the related cancer. Once infection has occurred, it may still be possible to protect against the development or progression of cancer, such as for HPV infection. A number of groups are now targeting the E6 and E7 proteins of HPV, as these so-called oncoproteins play a role in the transformation of infected cells into tumor cells. One such example utilizing DNA vaccines to target E6/E7 antigens in patients with high-grade cervical lesions due to HPV, resulted in CD8^+^ T cell responses [47].

Attempts to break tolerance to purely endogenous tumor antigens represented in large amounts on tumor cell surfaces such as alpha-fetoprotein (AFP), carcinoembryonic antigen (CEA) or prostatic acid phosphatase (PAP) have been more difficult to accomplish. Perhaps the most successful example has been Sipuleucel-T licensed in 2010 in the USA for prostate cancer [48]. The patient’s own dendritic cells are incubated *ex vivo* with an endogenous enzyme prostate acid phosphatase (PAP) and the immunostimulating agent GM-CSF; subsequently, the mixture is given back to the patient. For DNA vaccines, a veterinary cancer product is Oncept^TM^. It is a DNA vaccine encoding the human enzyme tyrosinase, and has been licensed for the treatment of melanoma, in dogs. The human tyrosinase differs from the canine version, enabling tolerance to be broken. In humans, a similar type of heterogenous tyrosinase vaccine, delivered with electroporation, showed increased CD8^+^ in 40% of the patients at the highest dose [49]. 

A clinical trial of DNA encoding a modified CEA resulted in some immune responses but unclear tumor reduction [29,50]. A clinical trial of a DNA vaccine encoding epitopes of Prostate Specific Acid Phosphatase (PSAP) linked to a fragment of tetanus toxin resulted in a doubling of the rate of rise of Prostate Specific Antigen (PSA, a marker for tumor cell growth) [51]. However a separate study of a DNA vaccine encoding rhesus Prostate Specific Antigen did not result in any change in the rate of rise of PSA levels [52].

DNA vaccines may have benefits for the development of idiotype-specific vaccines for B cell lymphomas because DNA vaccines could readily be made directed against the patient’s idiotype [53,54]. More recently, it has been shown in mice that a DNA vaccine could elicit cross-reactive anti-idiotype antibodies directed against human B cell lymphomas [55].

**Table 1:** Diseases for which DNA vaccines have entered clinical trials.

Infectious Diseases	Cancer	Other
Human immunodeficiency virus	B-cell lymphoma	Type I Diabetes
Influenza (Seasonal, Pandemic)	Prostate	Asthma
Malaria	Breast	
Hepatitis B	Melanoma	
Seasonal Acute Respiratory Syndrome	Ovarian	
Marburg	Cervical (Precancerous *In Situ*)	
Ebola	Hepatocellular	
Human Papilloma Virus (see Cancer)	Bladder	
West Nile Virus	Lung	
Dengue	Sarcoma	
Herpes Simplex Virus	Renal cell	
Measles	Lymphoplasmacytic lymphoma	
Cytomegalovirus	Colorectal	

Modified from Immunological reviews by Liu [2] with additions.

## 3. Conclusions

During the last few years, there has been immense progress in the field of DNA vaccines. This has been a result of new and better vectors, different types of delivery methods and devices, addition of immunologic adjuvants, and harnessing (or decreasing the activation of) the innate system, which is activated by the plasmid DNA itself, and can be further activated by encoded proteins. The combination of DNA vaccines with other vectors for heterologous prime-boost regimes, and selection of optimal diseases/antigens and vaccines also are important for making successful DNA vaccines. The present issue on “Research progress for gene-based vaccines” brings together primary data and up-to-date summaries of breakthroughs in using DNA plasmids for vaccines and immunotherapies.
